# War and Health Care Services Utilization for Chronic Diseases in Rural and Semiurban Areas of Tigray, Ethiopia

**DOI:** 10.1001/jamanetworkopen.2023.31745

**Published:** 2023-08-31

**Authors:** Tesfay Gebregzabher Gebrehiwet, Haftom Temesgen Abebe, Abraha Woldemichael, Kibrom Gebresilassie, Mache Tsadik, Akeza Awealom Asgedom, Girmatsion Fisseha, Kiros Berhane, Aregawi Gebreyesus, Yibrah Alemayoh, Measho Gebresilassie, Hagos Godefay, Hailay Abrha Gesesew, Solomon Tesfaye, Elias S. Siraj, Maru W. Aregawi, Afework Mulugeta

**Affiliations:** 1 School of Public Health, College of Health Sciences, Mekelle University, Mekelle, Tigray, Ethiopia; 2 School of Medicine, College of Health Sciences, Mekelle University, Mekelle, Tigray, Ethiopia; 3 Department of Biostatistics, Mailman School of Public Health, Columbia University, New York, New York; 4 Tigray Regional Health Bureau, Mekelle, Tigray, Ethiopia; 5 Research Centre for Public Health, Equity and Human Flourishing, Torrens University Australia, Adelaide, South Australia, Australia; 6 Diabetes Research Unit, Sheffield Teaching Hospitals and the University of Sheffield, Sheffield, United Kingdom; 7 Division of Endocrinology & Strelitz Diabetes Center, Eastern Virginia Medical School, Norfolk; 8 Global Malaria Program, World Health Organization, Geneva, Switzerland

## Abstract

**Importance:**

The war in Tigray, Ethiopia, has disrupted the health care system of the region. However, its association with health care services disruption for chronic diseases has not been well documented.

**Objective:**

To assess the association of the war with the utilization of health care services for patients with chronic diseases.

**Design, Setting, and Participants:**

Of 135 primary health care facilities, a registry-based cross-sectional study was conducted on 44 rural and semiurban facilities of Tigray. Data on health services utilization were extracted for patients with tuberculosis, HIV, diabetes, hypertension, and psychiatric disorders in the prewar period (September 1, to October 31, 2020) and during the first phase of the war period (November 4, 2020, to June 30, 2021).

**Main Outcomes and Measures:**

Records on the number of follow-up, laboratory tests, and patients undergoing treatment of the aforementioned chronic diseases were counted during the prewar and war periods.

**Results:**

Of 4645 records of patients with chronic diseases undergoing treatment during the prewar period, 998 records (21%) indicated having treatment during the war period. Compared with the prewar period, 59 of 180 individuals (33%; 95% CI, 26%-40%) had tuberculosis, 522 of 2211 (24%; 95% CI, 22%-26%) had HIV, 228 of 1195 (19%; 95% CI, 17%-21%) had hypertension, 123 of 632 (20%; 95% CI, 16%-22%) had psychiatric disorders, and 66 of 427 (15%; 95% CI, 12%-18%) had type 2 diabetes records, which revealed continued treatment during the war period. Of 174 records of patients with type 1 diabetes in the prewar period, at 2 to 3 months into the war, the numbers dropped to 10 with 94% decline compared with prewar observations.

**Conclusions and Relevance:**

This study found that the war in Tigray has resulted in critical health care service disruption and high loss to follow-up for patients with chronic disease, likely leading to increased morbidity and mortality. Local, national, and global policymakers must understand the extent and impact of the service disruption and urge their efforts toward restoration of those services.

## Key Points

**Question:**

What is the association of war with utilization of health care services among patients with chronic disease in Tigray, Ethiopia?

**Findings:**

In this cross-sectional study, of 4645 records of patients with chronic disease undergoing treatment retrieved in the prewar period, 998 (21%) received treatment during the war period. There was a dramatic decline of 80% of care for patients with type 1 diabetes.

**Meaning:**

The findings suggest that the war in Tigray has resulted in a dramatic disruption of the care for patients with chronic diseases, likely leading to increased morbidity and mortality from those conditions.

## Teaser

This cross-sectional study assesses the association of the war in Tigray, Ethiopia, with the utilization of health services for patients with chronic diseases.

## Introduction

Health care system disruption is a common consequence of war: a result of ignoring the declarations, resolutions, and statements of international humanitarian laws, the Geneva Convention, and the World Health Organization, by warring parties.^1,2^ Wars affect health and well-being of millions of adults and children.^3,4,5^ Forced displacements, migration, injury, disability and death of health care workforce, destruction of health infrastructure including lifesaving medical supplies, loss of electricity, means of communication, and water supply have been documented as the adverse impact of war leading to the collapse of health care systems.^6,7,8^ The deliberate targeting of the health care system is another cause of health service disruption with immediate and long-term implications.^9,10^ The interruption of routine treatment and follow-up, especially for those with chronic diseases, leads to increased morbidity and mortality.^11,12,13,14^ The war on Tigray, Ethiopia, which erupted on November 4, 2020, has led to the partial to complete destruction of the functions of all health facilities and pharmacy outlets of the region.^15,16^ In addition, ambulances, warehouses, and the pharmaceutical factory were destroyed.^17,18,19,20^ Reports from humanitarian and governmental organizations^17,18,19,20^ in Tigray indicated (1) pillaging of drugs, furniture, and medical equipment; (2) nonfunctionality of more than two-thirds of the health facilities; (3) displacement of a significant number of health care workers; and (4) the complete closure of the health posts.^9,10,17,18,19,20,21,22^ This resulted in severe disruption of the health services in the primary health care units.

Although there have been few reports,^15,18,19^ the association of the war with health services disruption for chronic diseases in primary health care units of Tigray has not been well documented. Therefore, this health care facility–based study aims to assess the association of the war with health service utilization, related to chronic diseases, and to compare the prewar and wartime of service in rural and semiurban health facilities in Tigray, Ethiopia.

## Methods

### Study Design and Ethical Approval

A registry-based cross-sectional study design was used to assess the health service utilization for chronic diseases and to compare services before and during the war. Data collection was conducted from July 3 to August 30, 2021. The data collected covered the period from September 1 to October 31, 2020, as prewar, while November 4, 2020, to June 30, 2021, as during the war. Ethical approval was obtained from the institutional review board of the College of Health Sciences, Mekelle University. Moreover, a support letter and letters of permission from the Tigray Health Bureau were obtained before the start of data collection. To use patient data from the registry, a waiver was granted by the ethic committee of Mekelle University. The report was organized using Strengthening the Reporting of Observational Studies in Epidemiology (STROBE) reporting guideline.

### Study Area and Period

Tigray is one of the 11 federal regions in Ethiopia. Tigray comprises 7 administrative zones that are further subdivided into a total of 93 *woredas* (districts) and 673 *Tabias* (subdistricts). The total population of Tigray in 2020 was estimated 7.3 million.^15^ Prior to the war, the public Health Sector of Tigray consisted of 2 tertiary hospitals, 14 secondary hospitals, 24 primary hospitals, 224 health centers, and 741 health posts. Thus, the physical access to basic health care services from both the public and private sectors was about 95%.^23^

### Sample Size and Sampling Technique

All zones of Tigray were included for this study except the Western zone since it was inaccessible due to occupation by hostile armed groups. Of the 84 districts in Tigray, 17 were excluded because of their urban setting. Of 67 rural and semiurban districts, total of 28 districts that were thought to accommodate a high load of patients with chronic diseases were purposively selected. Picking 2 health facilities randomly from each selected district, 56 of 135 health facilities (46 health centers and 10 primary hospitals) were included in the study. However, patient registers of 12 health centers were found to be completely destroyed and only 44 of the 56 health facilities were thus included in the analysis (eFigure in Supplement 1). Three types of data were gathered: (1) whether patients had follow-up visits, (2) whether they received laboratory services, and (3) whether they received treatment.

### Data Collection

The registration book of patients with tuberculosis, HIV, diabetes, hypertension, and psychiatric disorders who received health care services from selected facilities was reviewed. The data collectors visited health facilities and retrieved data from the reports and registration books.

A data extraction checklist was developed by the research team. Training was given to data collectors and supervisors on data extraction checklist and the objective of the survey. The variables (prewar, war period, months, follow-up, laboratory test, treatment received, and the selected type of chronic diseases) were clearly defined during the training. The confidentiality of patient data and anonymity was secured by using codes instead of personally identifiable information. Data on sex and age of patients were not available.

### Statistical Analysis

A descriptive analysis was conducted to compare health service delivery for chronic diseases during the prewar and war period. Follow-up of patients with tuberculosis, HIV, diabetes, hypertension, and psychiatric disorders, laboratory tests performed, and patients receiving treatment were described using frequency, percentages, bar graphs, and line graphs. We used Stata version 15 statistical software (StataCorp) to clean and analyze the data.

## Results

Of 44 health facilities included in this analysis, 30 health care centers and 4 primary hospitals were from the rural areas, and 4 health care centers and 6 primary hospitals were from the semiurban areas. There were 4645 records of patients with chronic diseases undergoing treatment during the prewar period. Compared with the prewar period, 840 of 4353 patients (19%; 95% CI, 18%-20%) were found at follow-up, and 998 of 4645 (21%; 95% CI, 20%-22%) received treatment during the war period. Follow-up and treatment services reduced by 81% (3513 of 4353) and 79% (3647 of 4645), respectively, during the war period compared with the prewar period (Table 1). The decline of patients’ flow was much higher in Central and Eastern zones with 95% (540 of 571) and 88% (1963 of 2239) reductions, respectively. Of all types of chronic diseases, records of patients with diabetes and hypertension receiving treatment during the war period were the lowest, with frequencies 66 of 427 (15%; 95% CI, 12%-18%) and 228 of 1195 (19%; 95% CI, 17%-21%) compared with the prewar period.

**Table 1.** Service Utilization of Patients by Type of Disease in Tigray, Ethiopia, Before and During the War

Chronic disease	Health care service delivered	Patients receiving services	
		Prewar period, No.	War period, No. (%) [95% CI]
Tuberculosis	Follow-up visit	145	42 (29) [21-36]
	Laboratory visit	94	34 (36) [26-46]
	Treatment	180	59 (33) [26-40]
HIV	Follow-up visit	1942	373 (19) [17-21]
	Laboratory visit	801	72 (9) [7-11]
	Treatment	2211	522 (24) [22-26]
Type 2 diabetes	Follow-up visit	439	60 (14) [11-17]
	Laboratory visit	234	19 (8) [4-11]
	Treatment	427	66 (15) [12-18]
Hypertension	Follow-up visit	1195	236 (20) [18-22]
	Treatment	1195	228 (19) [17-21]
Psychiatric disorders	Follow-up visit	632	126 (20) [17-23]
	Treatment	632	123 (19) [16-22]
Total	Follow up	4353	840 (19) [18-20]
	Laboratory visit	1129	125 (11) [9-13]
	Treatment	4645	998 (21) [20-22]

### Service Utilization of Patients With HIV 

**Figure 1.**
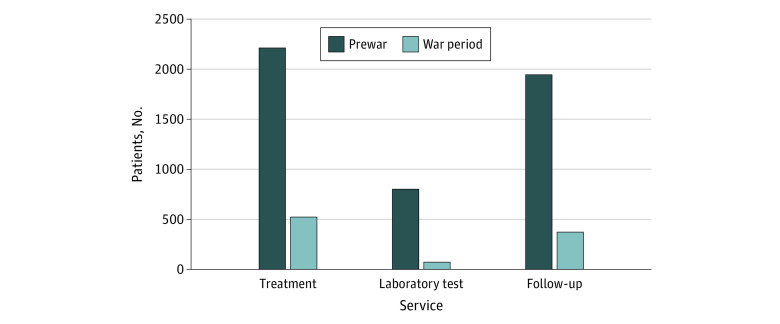
Service Utilization of People Living With HIV at Health Care Facilities in Tigray, Ethiopia, Before and During the War

The number of people living with HIV enrolled in antiretroviral therapy treatment services in the sampled health facilities dropped from 2211 prewar to 522 during the war period, indicating 76.4% reduction (Figure 1). The number of enrolled patients undergoing antiretroviral therapy who had clinical follow-up fell from 1942 prewar to 373 during the war period, representing 80% decrease.

### Service Utilization of Patients With Type 2 Diabetes

**Figure 2.**
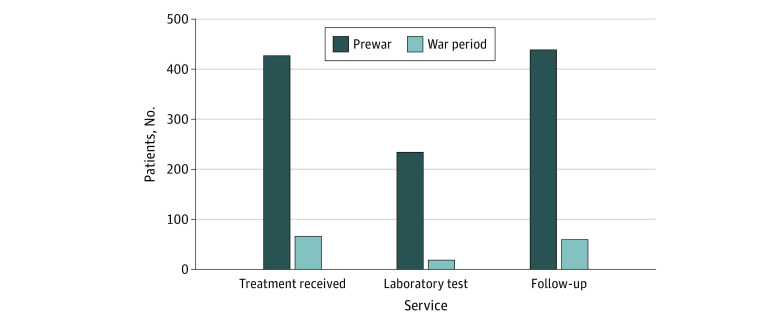
Service Utilization of Patients with Type 2 Diabetes at Health Care Facilities in Tigray, Ethiopia, Before and During the War

The records of 5 sampled primary hospitals showed only 15% (66 of 427) and 4% (19 of 234) of patients with type 2 diabetes received treatment and laboratory services during the war period, respectively (Figure 2). Follow-up services dropped from 439 before the war to 60 during the war period.

### Treatment of Patients With Type 1 Diabetes 

**Figure 3.**
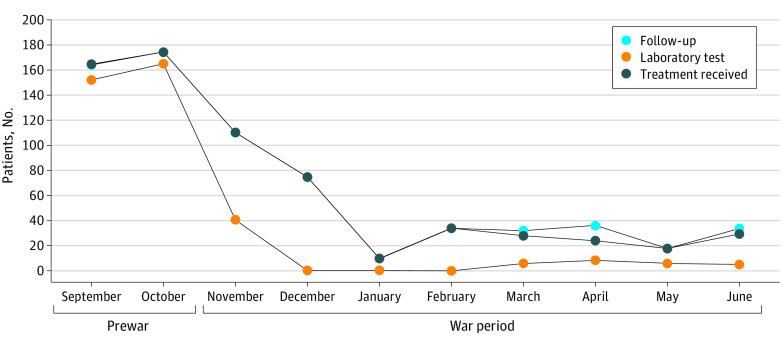
Changes in the Utilization of Services by Patients With Type 1 Diabetes Before and During the War

Before the war, as of October 2020, 174 patients with type 1 diabetes received treatment (Figure 3). At 2 to 3 months into the war (January 2021), the numbers dropped to observations between 10 at the lowest and 34 at the highest per month, showing a sustained decline of 80% to 94%.

In Hawzien Primary Hospital, 25 patients with tuberculosis, 398 with HIV, 260 with diabetes, 515 with hypertension, and 230 with psychiatric disorder received treatment before the war, while the numbers reduced to 2, 0, 5, 6, and 0, respectively, during the war period (Table 2).

**Table 2.** Service Utilization of Patients at Primary Hospital by Disease in Tigray, Ethiopia, Before and During the War

Chronic disease	Health care service delivered	Patients receiving services	
		Prewar period, No.	War period, No. (%)
Tuberculosis	Follow-up visit	25	2 (8)
	Laboratory visit	25	2 (8)
	Treatment	25	2 (8)
HIV	Follow-up visit	398	0 (0)
	Laboratory visit	398	0 (0)
	Treatment	398	0 (0)
Diabetes	Follow-up visit	260	5 (1.9)
	Laboratory visit	260	5 (1.9)
	Treatment	260	5 (1.9)
Hypertension	Follow-up visit	515	6 (1.2)
	Treatment	515	6 (1.2)
Psychiatric disorders	Follow-up visit	230	0 (0)
	Treatment	230	0 (0)
Total	Follow up	1428	13 (0.9)
	Laboratory visit	683	7 (1.0)
	Treatment	1428	13 (0.9)

## Discussion

The record of our study demonstrated that only 21% of patients with chronic diseases utilized health care services during the war period compared with prewar. This figure is less than a quarter of the Syrian refugees’ health care service utilization (84.7%) reported from a study in Jordan.^24^ Poor service utilization in conflict affected settings often reported due to the damage of health facilities and exodus of health care workers.^24,25,26^

Limitations and interruptions in the supply of medicines and resources has much affected the health care service utilization.^27,28^ The worst situation for patients with chronic disease due to service disruption observed in our study was similar to that reported from Myanmar.^29^ Like our findings, patients with chronic diseases in conflict-affected settings are subjected to the denial of access to optimal treatment or complete lack of treatment.^30,31^

The significant reduction in health care service utilization during the war period observed in our study is consistent with an earlier report from Ethiopia, where 91% of the studied health facilities^32^ and a recent report where 4 of 5 health facilities in Tigray^15^ ceased to provide services due to war. The physical destruction and looting of the health care facilities, presence of active ongoing conflict, lack of transportation, and flight of the health care workers were among the common reasons for poor health care services utilization.^26,29,33,34^ Delayed actions to provide responsiveness of health care services inhibit the functionality of the health facilities and to uplift the health care system, and weak coordination of donor agencies were the major threats that might lead to increased morbidity and mortality among patients with chronic diseases.^35,36^

The analysis indicated that almost half of the patients with chronic diseases missed their clinical follow-up and treatment during the first phase of the war. This finding is similar to that reported from studies in other conflict settings in low-income countries.^37,38,39^ The siege imposed on Tigray that the World Health Organization described as a de facto blockade could also have resulted in serious health care service disruption.^19,21,22,40,41^

The high reduction in clinical follow-up of patients from the Eastern and Central zones of Tigray observed in the record of our study is consistent with the higher proportion of health care facility destructions reported from these zones.^41^ Furthermore, the lengthy period of occupation by the invading troops and the displacement of the health care workers were crucial factors in the collapse of the health care system and the disruption of services.^19,21^ Thus, the cumulative impact of the war will have an implication of damaging the health of the Tigrayan population in the future with profound socioeconomic, psychosocial, and environmental consequences.

Unlike the marked decline in clinical follow-up of patients with diabetes and hypertension during the war period in our study, others from Iraq reported improved follow-up care of patients in camp-based public hospitals.^42^ The improvement in follow-up care might be related to ensuring the functionality of health care facilities and the retention of skilled health care professionals. The civil war in Syria also has provided a valuable lesson on the need to deliver appropriate health care services for patients with hypertension and diabetes during humanitarian crises.^43,44,45^ Compared with other conflict settings, the deliberate siege in Tigray was a unique experience hampering medical supplies from entering the community even by humanitarian agencies. On the other hand, the generous support to Syrian refugees by their neighboring countries supported them to access health care services via the facilitation of the International Organizations.^24,26,27^

The finding in our study was notable; of 174 patients with type 1 diabetes prewar, only 10 received treatment during the war, indicating loss to follow-up of 94%. Contrary to this finding, others reported an increase in access to service for patients with type 1 diabetes from 45% at baseline to 92% through the intervention of humanitarian assistance.^44^ This increased service access might be due to effort and appropriate humanitarian response that ensured improved and sustained availability of insulin for patients with type 1 diabetes in the mobile clinics.^44^ However, a study in 13 low- and middle-income countries reported the scarcity of insulin even in ordinary circumstances.^46^ The lack of insulin leads patients with type 1 diabetes to diabetic ketoacidosis, a lethal complication of insulin-dependent diabetes*.* Given the fact that lack of insulin can be fatal for those with type 1 diabetes, there are concerns that many, if not all, of those patients in our study who did not come for follow-up may have died without treatment. A 2016 study also indicated the increased probability of death among patients with type 1 diabetes due to the critical shortage of insulin supply.^9^

Per the registry, the missed follow-up and treatment of 67% of patients with tuberculosis and 76% of people living with HIV during the war period in our study was comparable with the earlier study reports from Tigray.^11,12,13^ Similarly, others reported the profound impact of loss to follow-up of patients with tuberculosis during the war period.^47^ Reduced attention to the health care needs of these populations, which is against humanitarian principles and medical ethics, can contribute to the high loss of follow-up of the patients that might end with risk of life-threatening conditions.^48^ The cessation of the critical role of health extension workers in mobilizing patients with tuberculosis and with HIV for care through house-to-house visits in the study context due to the war might have aggravated the disruption of health care services.^49^ Thus, the missing clinical follow-up and treatment of individuals with tuberculosis and HIV can lead to increased transmission of the disease and would pose the risk of treatment failure and risk of the development of multidrug-resistant tuberculosis in the index cases. Similarly, the likelihood of developing comorbidities among patients with HIV is very high.^50,51,52^

The displacement of civilians including the health care workforce in our study might also have contributed to the health care service disruption. A report from the study setting revealed that more than 2.5 million people were displaced due to war and remained without access to essential health services.^19^

Compared with the prewar period, the registry indicated only a fifth of patients with hypertension and diabetes utilized treatment during the war period. However, a war-related study among refugees in Middle East countries reported that patients with noncommunicable diseases had better services access at all levels of health care and better service utilization.^53^ This better access to health services might be related to reaching out to patients through United Nations humanitarian assistance and integrating the noncommunicable diseases health care service with other routine health care services at the primary care level.^54,55^ The limited donor commitment, poor distribution of pharmaceuticals, and poor financial allocation can aggravate the disruption of health services.^36^ Hypertension and diabetes are the most common risk factors for cardiovascular deaths and disabilities. Poor follow-up of patients with hypertension and diabetes are prone to develop stroke, myocardial infarction, blindness, and kidney disease, especially when they face 2 or more risk factors coexist.^56^

### Limitations

A limitation is that our study cannot determine causality due to the nature of its study design. In addition, record of some specific laboratory test results that might have indicated further insight (blood glucose for diabetes, sputum for tuberculosis, and HIV testing) were not retrieved due to the logistic reasons.

## Conclusion

The war on Tigray resulted in a devastating impact on the health care system of Tigray. Moreover, it caused a massive displacement of people, where the ability of the people to access the near-collapse health care system remained significantly affected. It is important to note that the negative impact of the war is likely to be only a fraction of the true extent of the consequence because the situation has worsened significantly after June 2021 due to the continued siege and blockade of Tigray and the resumption of fighting on several occasions. Today more than ever before, a significant number of patients with tuberculosis, HIV, diabetes, hypertension, psychiatric disorders, and other noncommunicable chronic diseases are either dying or experiencing high levels of traumatizing events due to disrupted health care services. Hundreds of patients with tuberculosis and HIV who have remained without treatment for months are at risk of developing drug resistance to the illnesses and irreversible complications that will lead to higher morbidity and mortality. In addition, the lack of access to essential medicines for chronic diseases and the absence of transport, banking, and communication services have put thousands of patients at risk of life-threatening situations. These facts call for urgent policy action to ensure the reestablishment of a fully functional health care system, protect and support the unpaid health workers serving in armed conflict zones, build the health care system’s ability to cope with both new and preexisting health care needs, and save the lives of thousands of patients before they develop devastating complications and death. This critical human rights concern requires an urgent response from members of the international community as well as public health and medical experts, researchers, scientists, professional associations, human rights activists, and consultants to save the life of the patients and improve their situation.

## Notes

Supplement 1. **eFigure. **Flow chart of selection of health facilities, September to October 2020 (prewar); November 2020 to June 2021 (war period)

Supplement 2.
Data Sharing Statement
