# Supplementary material for: War and Health Care Services Utilization for Chronic Diseases in Rural and Semiurban Areas of Tigray, Ethiopia
Source: JAMA Netw Open. 2023 Aug 31;6(8):e2331745. doi: 10.1001/jamanetworkopen.2023.31745 (PMC10472195; doi:10.1001/jamanetworkopen.2023.31745)
Supplement: Supplement 1. — eFigure. Flow chart of selection of health facilities, September to October 2020 (prewar); November 2020 to June 2021 (war period) [file jamanetwopen-e2331745-s001.pdf]

Supplemental Online Content

Gebrehiwet TG, Abebe HT, Woldemichael A, et al. War and health care services utilization for chronic diseases in rural and semiurban areas of Tigray, Ethiopia. *JAMA Netw Open*. 2023;6(8):e2331745. doi:10.1001/jamanetworkopen.2023.31745

eFigure. Flow chart of selection of health facilities, September to October 2020 (prewar); November 2020 to June 2021 (war period)

This supplemental material has been provided by the authors to give readers additional information about their work.

© 2023 Gebrehiwet TG et al. *JAMA Network Open.*

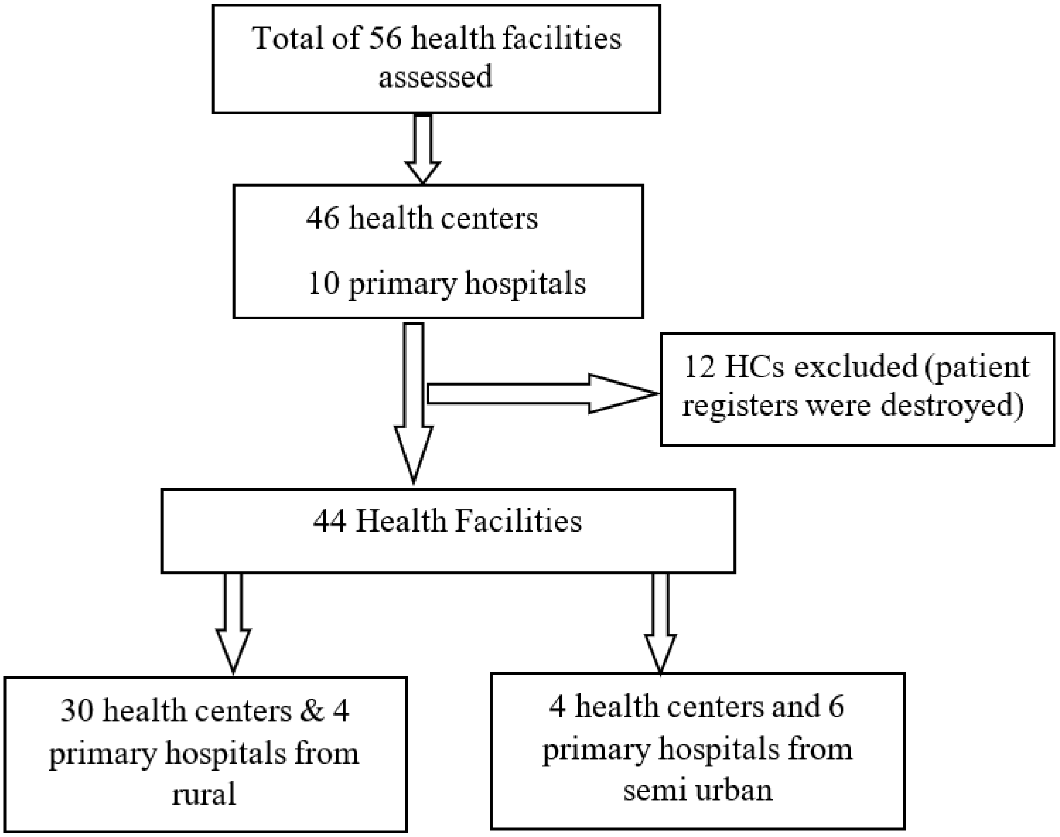

eFigure: Flow chart of selection of health facilities, September to October 2020 (prewar); November 2020 to June 2021 (war period)

© 2023 Gebrehiwet TG et al. *JAMA Network Open.*
