# Supplementary material for: War and Health Care Services Utilization for Chronic Diseases in Rural and Semiurban Areas of Tigray, Ethiopia
Source: JAMA Netw Open. 2023 Aug 31;6(8):e2331745. doi: 10.1001/jamanetworkopen.2023.31745 (PMC10472195; doi:10.1001/jamanetworkopen.2023.31745)
Supplement: Supplement 2. — Data Sharing Statement [file jamanetwopen-e2331745-s002.pdf]

Data Sharing Statement

Gebrehiwet. War and Health Care Services Utilization for Chronic Diseases in Rural and Semiurban areas of Tigray, Ethiopia. *JAMA Netw Open*. Published August 31, 2023. doi:10.1001/jamanetworkopen.2023.31745

Data
Data available: Yes
Data types: Deidentified participant data
How to access data: Available with the corresponding author and can be provided based on reasonable demand and permission that will be obtained from the regional health bureau. email address - tesfig@gmail.com
When available: With publication

Supporting Documents
Document types: Other (please specify)
Additional Information: Deidentified participant data
How to access documents: The documents are available with the author and Co-author. An email can be sent to tesfig@gmail.com or haftom.temesgen@mu.edu.et
When available: With publication

Additional Information
Who can access the data: The data can be available to researchers who would like to use the data for further work.
Types of analyses: Descriptive statistics
Mechanisms of data availability: With investigator support after approval of a proposal
